# Multicenter Hemodynamic Assessment of the LOT-CRT Strategy: When Does Combining Left Bundle Branch Pacing and Coronary Venous Pacing Enhance Resynchronization?: Primary Results of the CSPOT Study

**DOI:** 10.1161/CIRCEP.124.013059

**Published:** 2024-10-23

**Authors:** Marek Jastrzębski, Paul Foley, Badrinathan Chandrasekaran, Zachary Whinnett, Pugazhendhi Vijayaraman, Gaurav A. Upadhyay, Robert D. Schaller, Rafał Gardas, Travis Richardson, D’Anne Kudlik, Robert W. Stadler, Patrick Zimmerman, James Burrell, Robert Waxman, Richard N. Cornelussen, Jonathan Lyne, Bengt Herweg

**Affiliations:** First Department of Cardiology, Interventional Electrocardiology and Hypertension, Jagiellonian University, Medical College, Krakow, Poland (M.J.).; Wiltshire Cardiac Center, Great Western Hospital, Swindon, United Kingdom (P.F., B.C.).; Division of Cardiology, National Heart and Lung Institute, Imperial College, London, United Kingdom (Z.W.).; Division of Cardiology, Geisinger Heart Institute, Geisinger Commonwealth School of Medicine, Wilkes-Barre, PA (P.V.).; Center for Arrhythmia Care, Section of Cardiology, University of Chicago, Pritzker School of Medicine, IL (G.A.U.).; Section of Cardiac Electrophysiology, Cardiovascular Division, Department of Medicine, Hospital of the University of Pennsylvania, Philadelphia (R.D.S.).; Department of Electrocardiology and Heart Failure, Medical University of Silesia, Katowice, Poland (R.G.).; Division of Cardiovascular Medicine, Vanderbilt Heart, Nashville, TN (T.R.).; Medtronic, Minneapolis, MN (D.K., R.W.S., P.Z., J.B., R.W.).; Medtronic, Bakken Research Center, Maastricht, NL (R.N.C.).; Division of Cardiac Electrophysiology, Beacon Hospital (UCD), Dublin, Ireland (J.L.).; Division of Cardiology, University of South Florida Morsani College of Medicine and Tampa General Hospital, Tampa, FL (B.H.).

**Keywords:** bundle-branch block, cardiac resynchronization therapy, electrocardiography, hemodynamics, pacemaker

## Abstract

**BACKGROUND:**

Left bundle branch area pacing (LBBAP) may be an alternative to biventricular pacing (BVP) for cardiac resynchronization therapy (CRT). We sought to compare the acute hemodynamic and ECG effects of LBBAP, BVP, and left bundle-optimized therapy CRT (LOT-CRT) in CRT candidates with advanced conduction disease.

**METHODS:**

In this multicenter study, 48 patients with either nonspecific interventricular conduction delay (n=29) or left bundle branch block (n=19) underwent acute hemodynamic testing to determine the change in left ventricular pressure maximal first derivative (LV d*P*/d*t*_max_) from baseline atrial pacing to BVP, LBBAP, or LOT-CRT.

**RESULTS:**

Atrioventricular-optimized increases in LV d*P*/d*t*_max_ for LOT-CRT (mean, 25.8% [95% CI, 20.9%–30.7%]) and BVP (26.4% [95% CI, 20.2%–32.6%]) were greater than unipolar LBBAP (19.3% [95% CI, 15.0%–23.7%]) or bipolar LBBAP (16.4% [95% CI, 12.7%–20.0%]; *P*≤0.005). QRS shortening was greater in LOT-CRT (29.5 [95% CI, 23.4–35.6] ms) than unipolar LBBAP (11.9 [95% CI, 6.1–17.7] ms), bipolar LBBAP (11.7 ms [95% CI, 6.4–17.0]), or BVP (18.5 [95% CI, 11.0–25.9] ms), all *P*≤0.005. Compared with patients with left bundle branch block, patients with interventricular conduction delay experienced less QRS reduction (*P*=0.026) but similar improvements in LV d*P*/d*t*_max_ (*P*=0.29). Bipolar LBBAP caused anodal capture in 54% of patients and resulted in less LV d*P*/d*t*_max_ improvement than unipolar LBBAP (18.6% versus 23.7%; *P*<0.001). Subclassification of LBBAP capture (European Heart Rhythm Association criteria) indicated LBBAP or LV septal pacing in 27 patients (56%) and deep septal pacing in 21 patients (44%). The hemodynamic benefit of adding left ventricular coronary vein pacing to LBBAP depended on baseline QRS duration (*P*=0.031) and success of LBBAP (*P*<0.004): LOT-CRT provided 14.5% (5.0%–24.1%) greater LV d*P*/d*t*_max_ improvement and 20.8 (12.8–28.8) ms greater QRS shortening than LBBAP in subjects with QRS ≥171 ms and deep septal pacing capture type.

**CONCLUSIONS:**

In a CRT cohort with advanced conduction disease, LOT-CRT and BVP provided greater acute hemodynamic benefit than LBBAP. Subjects with wider QRS or deep septal pacing are more likely to benefit from the addition of a left ventricular coronary vein lead to implement LOT-CRT.

**REGISTRATION:**

URL: https://www.clinicaltrials.gov; Unique identifier: NCT04905290.

**WHAT IS KNOWN?**

- Patients with heart failure with intraventricular conduction delay and ischemic cardiomyopathy are less likely to respond to cardiac resynchronization therapy (CRT).
- Left bundle branch-optimized CRT reduces QRS duration more than left bundle branch area pacing or biventricular pacing alone.

**WHAT THE STUDY ADDS**

- In subjects with intact atrioventricular conduction but advanced conduction disease, Left bundle branch-optimized CRT and conventional biventricular pacing provided greater acute hemodynamic benefit than left bundle branch area pacing.
- Left bundle branch-optimized CRT reduced QRS duration more than left bundle branch area pacing or conventional biventricular pacing alone.
- Compared with patients with left bundle branch block, patients with intraventricular conduction disease experienced less QRS reduction during resynchronization pacing but similar improvements in left ventricular pressure maximal first derivative.
- The incremental value of left bundle branch-optimized CRT over left bundle branch area pacing to increase left ventricular pressure maximal first derivative was most pronounced in subjects with baseline QRS ≥171 ms and in patients where attempted left bundle branch area pacing resulted in only deep septal pacing.
- Anodal capture during left bundle branch area pacing decreased the hemodynamic benefit.

Left bundle branch area pacing (LBBAP) has emerged as a promising physiological pacing technique with potential applications in both bradyarrhythmia pacing^1^ and cardiac resynchronization therapy (CRT).^2^ When compared with biventricular pacing (BVP), LBBAP has been associated with improved mortality, reduced heart failure hospitalizations,^3^ and decreased incidence of both ventricular tachyarrhythmias and atrial fibrillation.^4^ However, proximal LBBAP may be limited in its ability to restore physiological activation of the lateral wall of the left ventricle (LV) in patients with conduction delay in the distal left bundle branch or its fascicles, the LV Purkinje network or myocardium.^5^ Moreover, in a substantial percentage of patients, direct left bundle (LB) capture cannot be achieved, resulting in LV septal pacing or even deep septal pacing (DSP), which may result in suboptimal correction of LV lateral wall delay.^6^

Conventional CRT, in the form of BVP via the LV coronary venous (LVcv) system combined with endocardial right ventricle (RV) pacing, has been shown in multiple randomized controlled trials to improve cardiac function, functional capacity, and survival while decreasing cardiac workload and hospitalizations.^7–9^ The limitations of conventional CRT include nonphysiological epicardial stimulation and the inability to effectively stimulate diseased tissue, such as myocardial scar (associated with latency, slow myocardial impulse propagation, and exit block).^10^ Further, its utilization is limited by unfavorable anatomy of the cardiac veins associated with suboptimal LVcv lead position (paraseptal/apical), lack of LVcv access, and diaphragmatic stimulation.^7^ Failure of conventional BVP-CRT to improve clinical outcomes is observed in up to one-third of patients who undergo CRT with BVP.^11,12^

Because LBBAP and BVP may be utilized as complementary resynchronization approaches that can be tailored to the individual patient or used in combination in the form of left bundle-optimized therapy (LOT) CRT, additional evidence is needed to determine when an LVcv lead should be added to LBBAP (or vice versa). The primary objective of this study was to compare the acute hemodynamic and electrocardiographic effects of 3 different resynchronization strategies (BVP, LBBAP, and LOT-CRT) in CRT candidates with intact atrioventricular conduction and advanced conduction disease.

## Methods

### Data Sharing

The data, analytic methods, and study materials are owned by the funder and will not be made available to other researchers.

### Study Design

The CSPOT study (Conduction System Pacing Optimized Therapy; ClinicalTrials.gov, REGISTRATION: URL: https://www.clinicaltrials.gov; Unique identifier: NCT04905290) was a prospective, multicenter, global study of LBBAP in CRT-indicated subjects. The primary objectives were to compare the acute hemodynamic and electrocardiographic effects of 3 distinctive approaches to resynchronization: (1) conventional CRT (BVP), (2) unipolar LBBAP only, bipolar LBBAP only, and (3) LOT-CRT, the combination of LBBAP and LVcv pacing. The study was conducted at 5 sites in Europe (2 in the United Kingdom, 1 in Ireland, and 2 in Poland) and 5 sites in the United States.

### Study Population

The study included subjects with a classic CRT indication,^13,14^ with a preference for subjects with intraventricular conduction disease (IVCD) and non-left bundle branch block. Left bundle branch block (LBBB) was defined using both the American Heart Association/American College of Cardiology/Heart Rhythm Society^15^ and Strauss criteria.^16^ Subjects with persistent or permanent atrial fibrillation or atrial flutter, second- or third-degree atrioventricular block, and isolated right bundle branch block with no additional conduction block were excluded. This study was performed according to the principles of the Declaration of Helsinki, and the study protocol was approved by the ethics committees of the respective hospitals. All subjects provided written informed consent before investigation.

### Study Protocol

Subjects underwent a baseline visit (enrollment, demographics, medical history, and baseline measurements, including echocardiographic evaluation). Thereafter, all participants received CRT implantation according to standard clinical practice, with additional LBBAP lead placement. The LBBAP lead (model 3830, 69 cm; Medtronic, Inc, Minneapolis, MN) was placed according to previously described methodology. Implantation of a quadripolar LVcv lead targeted the posterior-lateral or lateral branches of the LVcv when possible. In CRT-D subjects, a standard RV lead was positioned in the RV apex. A standard right atrial lead was positioned to maintain constant atrial rate during the protocol. Simultaneous acute hemodynamic and electrophysiological measurements were performed during various pacing configurations.

### Pacing and Recording Equipment

An external pacemaker (model 5388; Medtronic, Inc) provided atrial overdrive pacing (≈10 beats/minute above intrinsic rate) to maintain a constant heart rate. The atrial pacing also triggered the EP acquisition device (EP Tracer; Schwarzer-Cardiotek GMBH, Germany), which delivered single- or dual-site ventricular pacing (RV, LVcv, or LBBAP) with a programmable atrioventricular delay. The EP acquisition device recorded all physiological signals at 1 kHz (ie, intracardiac electrograms, 12-lead ECG, and LV pressure).

### Pacing Protocol

Selection of the best LVcv electrode on the quadripolar lead was selected by the physician, considering anatomic location, capture thresholds, absence of phrenic stimulation, and measurement of dyssynchrony (ie, the standard deviation of activation times during BVP from an array of 40 skin electrodes surrounding the thorax).^17^ A default atrioventricular delay was calculated as 70% of the interval between an atrial-paced event and the earliest QRS activation on the 12-lead ECG (Figure S1).^18^ A sweep of 5 atrioventricular delays was tested for each pacing configuration: default atrioventricular delay and atrioventricular delays 30 and 60 ms shorter and longer than the default atrioventricular delay. Each pacing configuration and atrioventricular delay was repeated 4×. Pacing configurations were measured in the following order: BVP, unipolar LBBAP, bipolar LBBAP, and LOT-CRT. The interventricular pacing delay (for BVP and LOT-CRT configurations) was set to zero for all configurations. To ensure pacing capture, pacing output was set at threshold plus 2 mA.

### Electrocardiographic Measurements

QRS duration was measured from the pacing artifact to the end of the QRS complex on the standard 12-lead ECG (Figure S1). For each pacing configuration, electrocardiographic response was defined by the absolute reduction in paced QRS duration relative to intrinsic activation. The greatest shortening in QRS duration was then estimated from a quadratic curve fit to the results observed at each atrioventricular delay.

For each patient, conduction disease classification, LBBAP capture classification, and the presence of anodal capture were blindly adjudicated, as follows.

#### Conduction Disease Classification

LBBB was defined via strict application of both the American Heart Association/American College of Cardiology/Heart Rhythm Society and Strauss criteria. Subjects not meeting both LBBB criteria were designated as IVCD.

#### LBBAP Capture Classification

LBBAP capture classification was based on the European Heart Rhythm Association clinical consensus statement.^6^ Successful placement of the LBBAP lead, suggesting early LV activation, was determined by a paced QRS complex with an *r*′ in lead V_1_. In a subset of subjects, suboptimal lead placement or advanced left conduction system disease resulted in absent *r*′ in lead V_1_ and broad QRS complexes, which was classified as DSP. We used LBBAP to refer to pacing from the LBBAP location. However, to describe specific capture types, we used the terms successful LBBAP and DSP.

#### Detection of Anodal Stimulation

LBBAP bipolar stimulation and LOT-CRT pacing (which included LBBAP bipolar stimulation) had the potential to capture at the ring (anode) of the 3830 lead. We assessed anodal capture by observing morphology changes in 12-lead ECGs or electrograms when comparing unipolar and bipolar LBBAP (Figure S2). The electrogram recorded from the RV apical lead was used to assess local RV activation time.

### Measurements: Hemodynamics

The LV pressure catheter (Micro-Cath, Millar, TX) was inserted into the LV cavity via the femoral artery, allowing continuous calibrated recording of LV pressure. LV pressure maximal first derivative (LV d*P*/d*t*_max_) was calculated offline using custom software (MATLAB R2023b; MathWorks, Inc). For each combination of pacing configuration and atrioventricular delay, LV d*P*/d*t*_max_ was first recorded during AAI pacing as a control, followed by alternating between DDD and AAI pacing 4× (10–15 beats each). This yielded 8 transitions, used to reduce variability in the LV d*P*/d*t* measurements, as shown by Pabari et al.^19^ Beats with ventricular or supraventricular extrasystoles and the subsequent beats were excluded.^20^ The percentage change in LV d*P*/d*t*_max_ from AAI to DDD or DDD to AAI was calculated for each of the 8 transitions. The greatest LV d*P*/d*t*_max_ improvement was estimated from a quadratic curve fit to the median percentage change calculated for each of the 5 atrioventricular delays.

### Statistical Analysis

Analysis included all subjects that completed the acute pacing protocol, regardless of whether DSP or successful LBBAP was achieved. Descriptive statistics were used to summarize baseline and procedural characteristics. Qualitative variables were summarized as frequencies and percentages, and continuous variables were summarized using means and standard deviations. The main objectives of comparing the hemodynamic and electrocardiographic effects between each pair of pacing configurations were evaluated using paired *t* tests. The Holm-Bonferroni method was used to adjust *P* values for multiple comparisons separately for primary hemodynamic and electrocardiographic responses. For subgroup analyses, mixed-effect models were fit with fixed effects for pacing configuration, group membership, and their interaction (to test whether the relative effects of individual pacing configurations differed between groups). A random intercept at the patient level was used to account for patient-level variability. The subgroup analyses by IVCD versus LBBB, ischemic cardiomyopathy (ICM) versus non-ICM (NICM), and baseline QRS duration ≥171 versus <171 were prespecified. All *P* values are based on a 2-sided significance level of 0.05. No adjustments were made for multiple testing other than those described for the main objectives. Analysis was conducted in R, version 4.2.3 (www.R-project.org), using the nlme^21^ and tidyverse^22^ packages.

## Results

### Patient Characteristics

Of the 60 subjects enrolled in the CSPOT study, 5 subjects were excluded before the procedure start (the patient or physician decided to withdraw) and 7 were excluded during the procedure (4 because of temporary atrioventricular block, 2 because the patient was unable to endure the length of the procedure, and 1 inability to place an LVcv lead). Table 1 shows that the 48 remaining subjects represented a heterogeneous CRT-indicated population. Patients were predominantly male (67%), nearly one-third had ICM, and the LV ejection fraction was 30.6%. A total of 40% were centrally adjudicated as LBBB while 60% had IVCD, as was intended by the inclusion criteria. Two subjects who did not undergo RV-defibrillation lead implantation were paced from the LV-only rather than BVP and were included in the BVP data set.

**Table 1.**
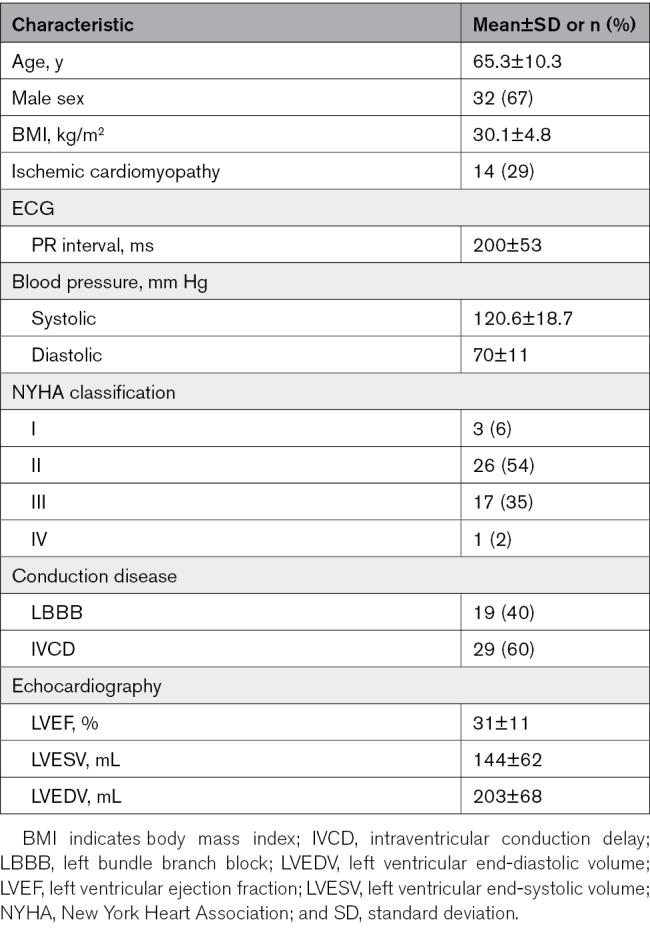
Subject Demographics

### Hemodynamic and Electrocardiographic Effects

**Figure 1.**
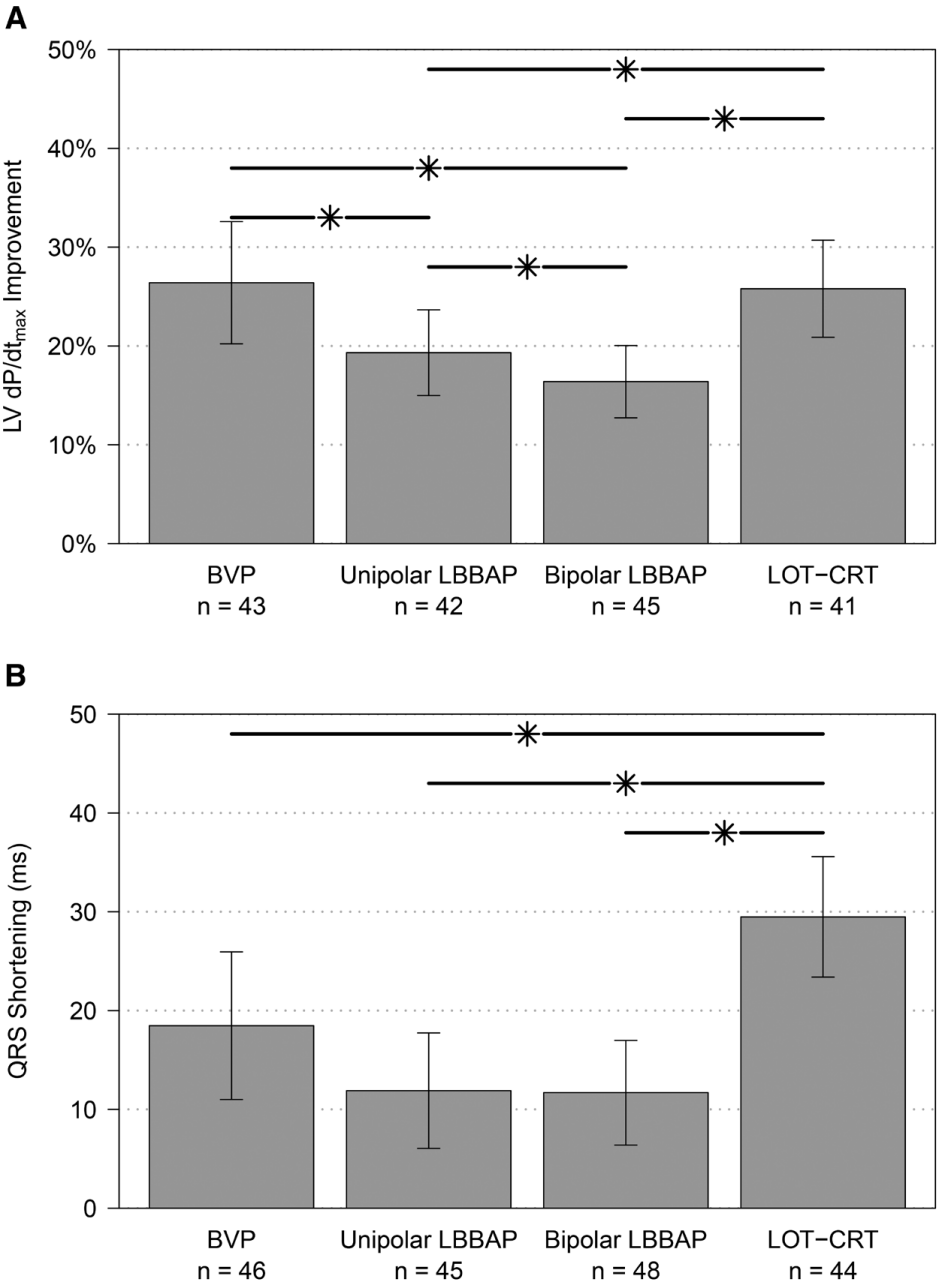
**Comparison of acute responses from different pacing configurations. A.** Left ventricular pressure maximal first derivative (LV d*P*/d*t*_max_) increase; **B.** QRS-duration shortening. Displayed are means±95% CIs. *P≤0.005. Left bundle branch area pacing (LBBAP), including deep septal pacing. BVP, biventricular pacing; and LOT-CRT, left bundle branch-optimized cardiac resynchronization therapy.

Mean baseline LV d*P*/d*t*_max_ was 846 mm Hg/s (Table 2), indicative of moderately depressed cardiac contractile function (54% of subjects presented with New York Heart Association class II). Figure 1A displays LV d*P*/d*t*_max_ improvement for each pacing configuration relative to AAI. BVP resulted in a 26.4% (95% CI, 20.2%–32.6%) improvement, similar to LOT-CRT (25.8% [95% CI, 20.9%–30.7%]; *P*=0.45). Both BVP and LOT-CRT resulted in larger improvement than either unipolar LBBAP (19.3% [95% CI, 15.0%–23.7%]) or bipolar LBBAP (16.4% [95% CI, 12.7%–20.0%]; *P*<0.001 for all except *P*=0.005 for BVP versus unipolar LBBAP), and unipolar LBBAP resulted in larger improvement than bipolar LBBAP (*P*=0.035).

**Table 2.**
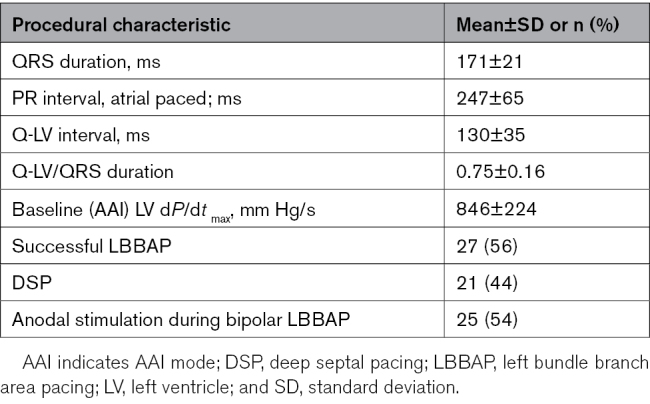
Procedural Characteristics

Mean baseline QRS duration was 171±21 ms (Table 2). Figure 1B displays QRS-duration shortening when compared with baseline. Shortening in paced QRS duration was greatest in the LOT-CRT configuration (29.5 [95% CI, 23.4–35.6] ms), compared with BVP (18.5 [95% CI, 11.0–25.9] ms; *P*=0.005), LBBAP bipolar (11.7 [95% CI, 6.4–17.0] ms; *P*<0.001), and LBBAP unipolar (11.9 [95% CI, 6.1–17.7] ms; *P*<0.01). There was no statistically significant difference in QRS shortening between BVP and either unipolar LBBAP (*P*=0.090) or bipolar LBBAP (*P*=0.090).

### Subgroup Analysis

#### LBBB Versus IVCD Classification

**Figure 2.**
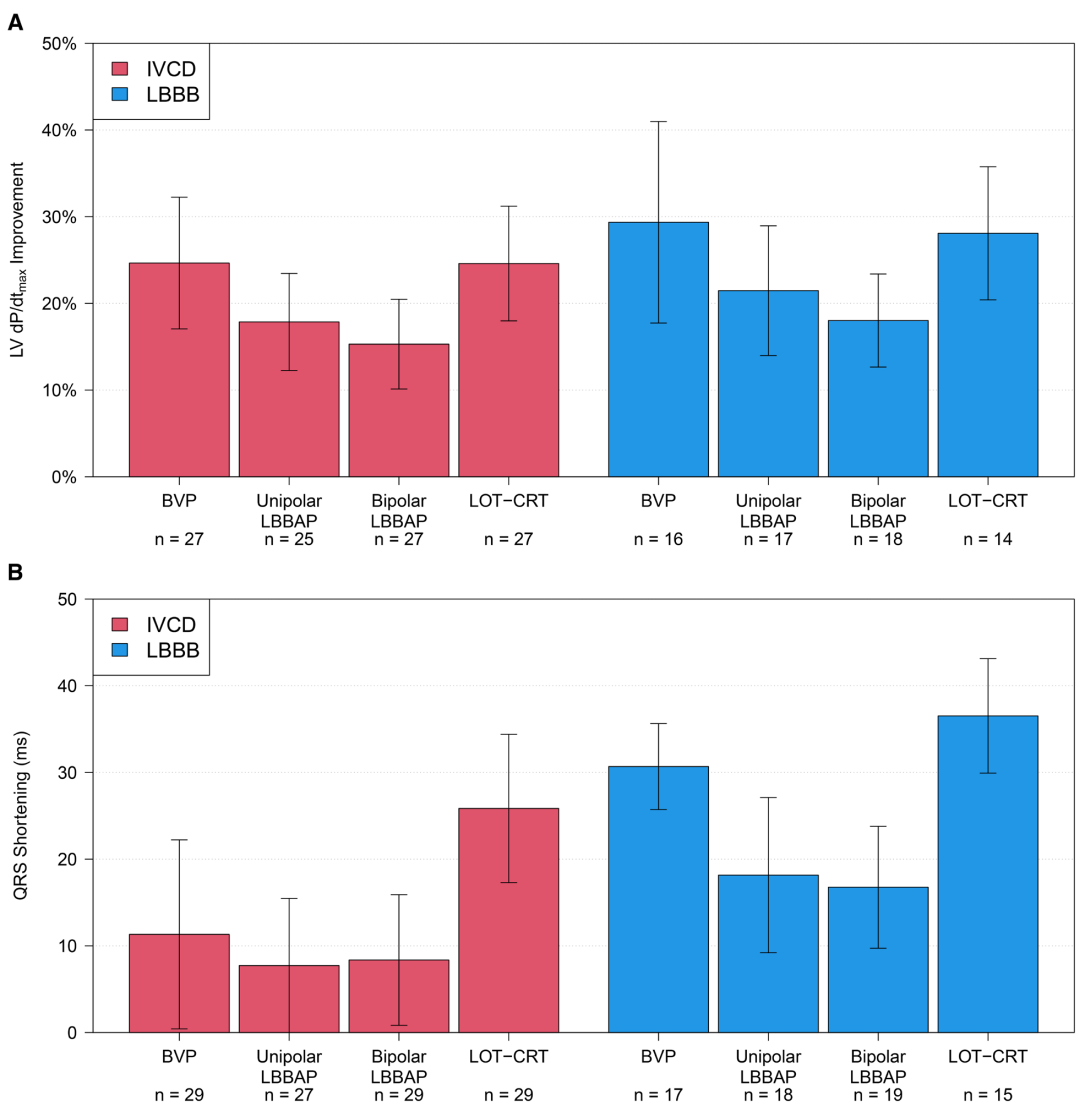
**Subgroup analysis according to conduction disease: intraventricular conduction disease (IVCD; red) or left bundle branch block (LBBB; blue). A**, Left ventricular pressure maximal first derivative (LV d*P*/d*t*_max_) increase (mean and 95% CIs), comparison between groups: *P*=0.29, comparison of relative effects of pacing configurations: *P*=0.79 and **B**, QRS-duration shortening (absolute decrease from baseline), comparison between groups: *P*=0.026, comparison of relative effects of pacing configurations: *P*=0.21. Left bundle branch area pacing (LBBAP), including deep septal pacing. BVP, biventricular pacing; IVCD, intraventricular conduction delay; and LOT-CRT, left bundle branch-optimized cardiac resynchronization therapy.

To analyze the impact of underlying conduction disease on treatment effect, we compared patients with LBBB (40%) to those with IVCD (60%; Figure 2). The improvement of LV d*P*/d*t*_max_ was similar between patients with LBBB or IVCD (*P*=0.29), and the relative effects of individual pacing configurations on LV d*P*/d*t*_max_ were similar between groups (*P*=0.79). Reduction in QRS duration was more pronounced among subjects with LBBB than in those with IVCD (*P*=0.026), but the relative effects of individual pacing configurations on the reduction of QRS duration were similar between groups (*P*=0.21).

#### Successful LBBAP Versus DSP

**Figure 3.**
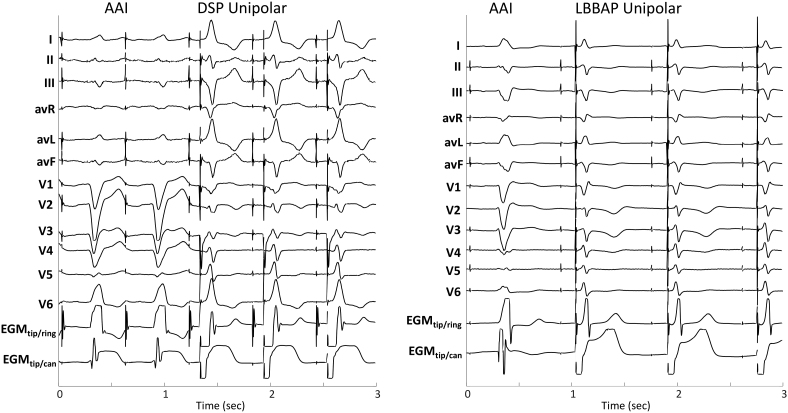
**Classification of capture types: Deep septal pacing (DSP) and successful left bundle branch area pacing (LBBAP).** DSP (left panel), LBBAP (right panel). AAI indicates AAI mode; and EGM, electrogram.

Placement of the LBBAP lead to achieve conduction system capture proved to be challenging in this patient population: successful LBBAP was achieved in 56% of patients, and DSP capture occurred in the remaining 44% of the patients (DSP was observed in at least 1 patient at each center). For example, recordings from DSP (left) and successful LBBAP (right) are shown in Figure 3. Lead V6 R-wave peak time was statistically significantly shorter with successful LBBAP (82 [95% CI, 74–89] ms) than with DSP (103 [95% CI, 91–115] ms; *P*<0.005).

**Figure 4.**
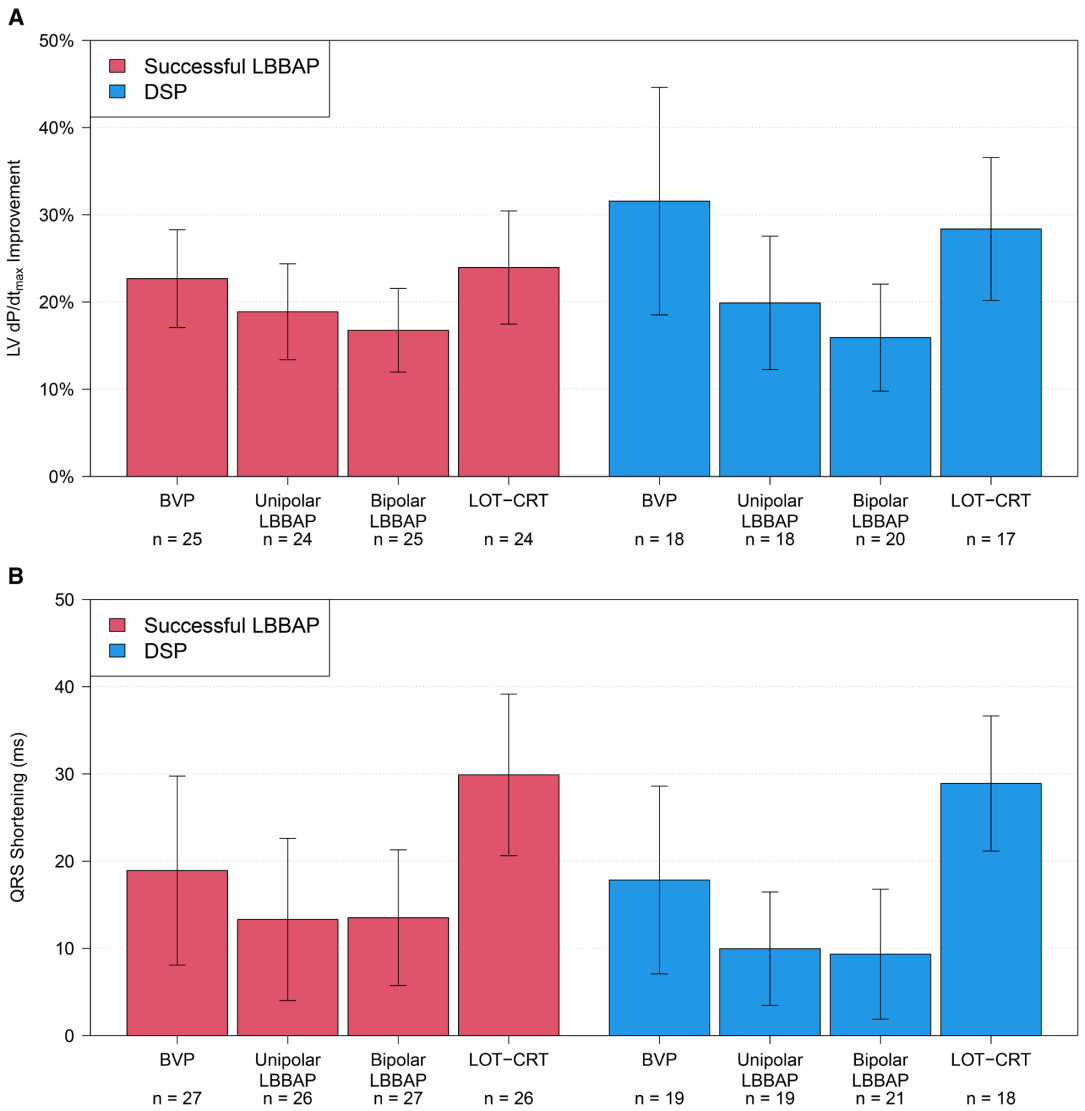
**Subclassification according to successful left bundle branch area pacing (LBBAP; red) or deep septal pacing (DSP; blue). A**, Left ventricular pressure maximal first derivative (LV d*P*/d*t*_max_) increase (mean and 95% CIs), comparison between groups: *P*=0.40, comparison of relative effects of pacing configurations: *P*<0.005 and **B**, QRS-duration shortening, comparison between groups: *P*=0.53, comparison of relative effects of pacing configurations: *P*=0.80. BVP, biventricular pacing; and LOT-CRT, left bundle branch-optimized cardiac resynchronization therapy.

The differences in acute contractile and electrocardiographic response between patients with successful LBBAP versus DSP are summarized in Figure 4. Overall, improvement of LV d*P*/d*t*_max_ (Figure 4A) was similar between successful LBBAP and DSP groups (*P*=0.40). However, the relative effects of individual pacing configurations differed between groups (*P*<0.005).

The improvement of LV d*P*/d*t*_max_ with LOT-CRT, relative to unipolar LBBAP, was more pronounced in patients with DSP (12.0% [95% CI, 6.2%–17.8%]) when compared with patients with successful LBBAP (5.1% [95% CI, 0.2%–9.9%]). Figure 4B presents the analogous results for reduction in paced QRS duration. Overall, QRS reduction was similar between successful LBBAP and DSP groups (*P*=0.53), and the relative effects of individual pacing configurations were similar between groups (*P*=0.80).

#### Baseline QRS Duration

**Figure 5.**
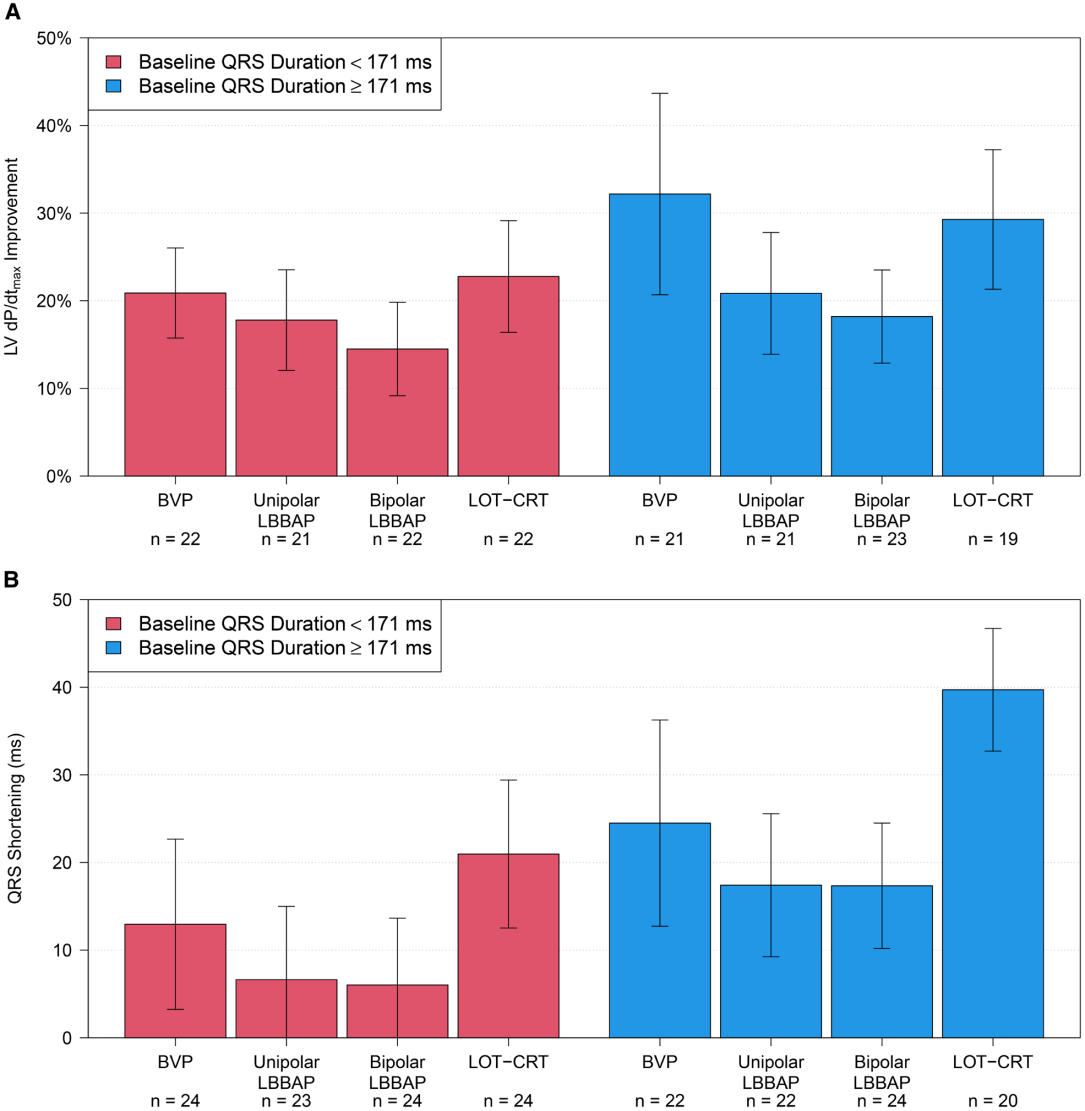
**Subclassification according to baseline QRS <171 ms (red) or ≥171 ms (blue). A**, Left ventricular pressure maximal first derivative (LV d*P*/d*t*_max_) increase (mean and 95% CIs), comparison between groups: *P*=0.12, comparison of relative effects of pacing configurations: *P*=0.031 and **B**, QRS-duration shortening, comparison between groups: *P*=0.014, comparison of relative effects of pacing configurations: *P*=0.47. Left bundle branch area pacing (LBBAP), including deep septal pacing. BVP includes biventricular pacing; and LOT-CRT, left bundle branch-optimized cardiac resynchronization therapy.

The mean baseline QRS duration of 171 ms was used to separate patient groups with narrower QRS (<171 ms) and wider QRS (≥171 ms; Figure 5). Overall, improvement of LV d*P*/d*t*_max_ (Figure 5A) was similar between patient groups (*P*=0.12). However, the relative effects of individual pacing configurations differed between groups, *P*=0.031. The improvement of LV d*P*/d*t*_max_ with LOT-CRT relative to unipolar LBBAP was more pronounced in patients with a wider baseline QRS ≥171 ms (10.4% [95% CI, −4.9% to 15.9%]) when compared with a patients with a baseline QRS duration <171 ms (5.5% [95% CI, 0.3%–10.8%]). Likewise, the improvement of LV d*P*/d*t*_max_ with BVP, relative to unipolar LBBAP, was more pronounced in patients with a wider baseline QRS ≥171 (14.1% [95% CI, 8.7–19.5%] ms) when compared with patients with a baseline QRS duration <171 (3.6% [95% CI, −1.6% to 8.9%] ms).

Figure 5B presents the corresponding results for reduction in QRS duration. Overall, QRS reduction was larger in patients with a baseline QRS duration ≥171 ms when compared with patients with baseline QRS <171 ms (*P*=0.014), but the relative effects of individual pacing configurations were similar between the groups (*P*=0.47).

#### Benefit of Adding LVcv Pacing to LBBAP (LOT-CRT)

**Figure 6.**
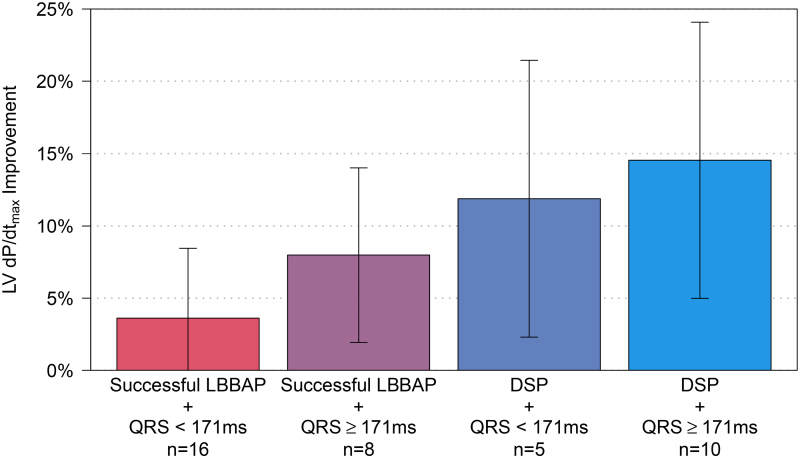
**Acute hemodynamic benefit of adding a left ventricular coronary vein lead to unipolar left bundle branch area pacing (LBBAP) pacing in 4 subgroups.** Each bar represents the left ventricular pressure maximal first derivative (%LV d*P*/d*t*_max_) improvement (mean and 95% CIs) between left bundle branch-optimized cardiac resynchronization therapy and unipolar LBBAP. DSP includes deep septal pacing.

To quantify the benefit of LOT-CRT compared with unipolar LBBAP, a post hoc analysis was conducted. Figure 6 shows the difference in LV d*P*/d*t*_max_ between LOT-CRT and unipolar LBBAP in 4 groups of subjects based on successful LBBAP versus DSP and baseline QRS duration <171 versus ≥171 ms. The largest benefit of adding LVcv pacing (=LOT-CRT) was observed in subjects with a baseline QRS ≥171 ms and DSP only, where LOT-CRT provided 14.5% (5.0%–24.1%; *P*<0.01) greater LV d*P*/d*t*_max_ improvement and 20.8 ms (12.8–28.8 ms; *P*<0.01) shorter QRS duration than unipolar LBBAP.

#### Anodal Stimulation

Twenty-five subjects (54%) had both anodal and cathodal stimulation during bipolar LBBAP, while 21 (46%) had only cathodal stimulation. An example of the surface electrocardiographic signature and the effect on the local RV electrogram of anodal capture is depicted in Figure S2. Bipolar LBBAP was associated with a statistically significant reduction in LV d*P*/d*t*_max_ improvement compared with unipolar LBBAP in patients with anodal stimulation (18.6% versus 23.7%; *P*<0.001) but similar improvement in patients without anodal stimulation (15.2% versus 14.2%; *P*=0.92). Further, in patients with anodal stimulation, bipolar LBBAP shortened RV activation time compared with unipolar LBBAP by 13.9 ms (95% CI, 8.4–19.5 ms; *P*<0.001). However, bipolar and unipolar LBBAP produced similar QRS duration shortening regardless of whether anodal stimulation was present. Anodal capture was similarly prevalent in subjects with DSP (57%; 12/21 patients) and successful LBBAP (52%; 13/25 patients; *P*=0.96).

#### Ischemic Cardiomyopathy

ICM was reported in 14 of 48 patients (29%) and associated with diminished improvement of LV d*P*/d*t*_max_ (*P*=0.004) and marginally worse shortening in QRS duration (*P*=0.06; Figure S3). However, for both outcomes, the relative effects of individual pacing configurations were similar between patients with and without ICM (*P*=0.95 for LV d*P*/d*t*_max_ and *P*=0.81 for QRS duration shortening).

## Discussion

We evaluated the acute hemodynamic and electrocardiographic effects of 3 different resynchronization strategies in a group of CRT candidates with intact atrioventricular conduction but advanced conduction disease. LOT-CRT achieved narrower QRS duration than LBBAP and BVP. LOT-CRT and BVP similarly improved LV d*P/*d*t*_max_ compared with LBBAP alone. Patients with IVCD experienced less QRS shortening than patients with LBBB, but the relative LV d*P*/d*t*_max_ response to the different pacing strategies was similar for IVCD and LBBB. The incremental value of LOT-CRT over LBBAP to improve LV d*P*/d*t*_max_ was most pronounced in patients with a baseline QRS ≥171 ms and DSP capture type. Although the improvements of LV d*P*/d*t*_max_ and QRS reduction were muted in patients with ICM, the relative performance of the 3 resynchronization strategies was similar in patients with ICM versus NICM. Finally, anodal capture during bipolar LBBAP resulted in a statistically significantly diminished LV d*P*/d*t*_max_ and earlier RV activation but no change in QRS duration when compared with unipolar LBBAP.

### LOT-CRT Versus BVP and LBBAP

LOT-CRT provided the best paced QRS reduction across all tested pacing configurations, which is confirmatory to observational studies published to date.^23,24^ The study by Jastrzebski et al^23^ however, attempted LOT-CRT only in subjects with a suboptimal paced QRS reduction after LBBP. In a patient population of NICM and LBBB, Parale et al^24^ found that QRS duration reduction with LOT-CRT was numerically larger but not statistically significantly different compared with LBBAP. In patients with IVCD, Chen et al^25^ showed superior (acute) electric synchrony and superior clinical outcomes with LOT-CRT versus BVP. In our study, the QRS reduction benefit of LOT-CRT over BVP was not corroborated by improved contractile function: LOT-CRT and BVP produced equivalent improvements in LV d*P*/d*t*_max_ in the overall cohort (Figure 1) as well as in the subpopulations of patients with LBBB and IVCD (Figure 2). We note, however, that LOT-CRT pacing in this study required bipolar LBBAP, which resulted in anodal stimulation in 54% of subjects and may have been detrimental hemodynamically. Further, our discrepant electric and contractile response might have been due to the small group size, the relatively large number of patients with ICM (Figure S3), or DSP capture (Figure 4).

### BVP Versus LBBAP

In our cohort, the acute hemodynamic benefit of BVP exceeded that of LBBAP, likely due to the high proportion of DSP. In patients with DSP, BVP improved LV d*P*/d*t*_max_ by 15.8% more than LBBAP, while this difference was only 3.8% in subjects with successful LBBAP (Figure 4). A recent study by Curila et al^26^ showed no difference in acute hemodynamic benefit (ie, systolic blood pressure) between LV septal pacing and BVP.

In comparison to previously published data,^27–29^ BVP pacing in our study provided a relatively large increase in LV d*P*/d*t*_max_ over baseline AAI pacing (26.4%), which may be attributed to good anatomic LVcv lead placement (evidenced by a 75% QLV/QRSd),^30^ selection of the best quadripolar electrode, and individual atrioventricular optimization. In contrast to our results, Liang et al^31^ found statistically significant larger LV d*P*/d*t*_max_ improvement with LBBAP (37%) than with BVP (32%) in a cohort predominantly consisting of patients with NICM and typical LBBB. They claimed LB capture was confirmed in all patients. Unlike other clinical studies,^32–34^ our acute hemodynamic study found no advantage of LBBAP over BVP, which may be explained by our high prevalence of DSP, ICM, and long baseline QRS duration.

### Prevalence of DSP

Failure to achieve LB capture and broad QRS complex (DSP) occurred in 44% of subjects, distributed over all study centers, which raises concerns and is more prevalent than reported thus far by others.^35^ Prior observational studies have generally reported higher rates of successful LBBAP or LV septal pacing, perhaps because patients had predominantly NICM and LBBB, and less rigorous evaluation of LB capture, thus, underreporting treatment failures (typical for observational studies).^36,37^ Our study data showed similar successful LBBAP in subjects with IVCD versus LBBB or ICM versus NICM. Our high prevalence of DSP is also unlikely to have resulted from shallow septal lead placement (anatomic DSP) because anodal capture occurred in 57% of subjects with DSP and 52% of subjects with successful LBBAP. Further, in the present study DSP clearly provided substantial hemodynamic benefit (Figure 4). This has been reported by other investigators as well.^38,39^ Another potential explanation is a lack of a healthy conduction system at LBBAP septal depth (functional DSP). Despite obtaining capture of the proximal left conduction system, slow impulse propagation in the distal conduction system and latency may result in a QRS morphology more consistent with DSP due to dominance of simultaneous RV activation. Although more in-depth investigation is warranted, these subjects may have more severe electric uncoupling by septal fibrosis resulting in more conduction delay in the distal left bundle branch fascicles and Purkinje network as well as impaired cell-to-cell conduction in the myocardium.^23,24^ Recent anatomic advancements indicate an ubiquitous presence of fasciculo ventricular pathways,^40^ potentially providing more anatomic locations to capture the central conduction system for LV resynchronization.

### Adding LVcv Pacing to LBBAP (LOT-CRT)

A primary objective of this study was to identify patients who are most likely to benefit from a LOT-CRT approach. As outlined in Figure 4, subjects with DSP capture benefited from the added LVcv lead: LOT-CRT provided a 12% improvement in LV d*P*/d*t*_max_ and a 15 ms QRS reduction when compared with unipolar DSP. As outlined in Figure 5, patients with a wider baseline QRS (≥171 ms) also benefited from the added LVcv lead. Larger baseline QRS duration might indicate more complex conduction disease and, therefore, a broader resynchronization strategy might be warranted.^41^ The combination of DSP and wider baseline QRS (≥171 ms) resulted in maximal supplementary benefit from the addition of an LVcv lead to an LBBAP lead (Figure 6).

Subjects with ICM had statistically significantly reduced LV d*P*/d*t*_max_ response and nearly statistically significantly reduced QRS reduction response compared with subjects with NICM to all 3 resynchronization approaches (Figure S3) and may, therefore, benefit from the addition of an LVcv lead to achieve a clinical response.^42,43^

### Anodal Capture During LBBAP

LBBAP can be delivered in unipolar or bipolar polarity, and bipolar polarity may result in anodal capture. In this study, subjects were retrospectively adjudicated for anodal capture based on differences between electrocardiograms and electrograms recorded during unipolar and bipolar LBBAP and bipolar LBBAP amplitude threshold testing. Anodal capture from the 3830 lead was noted to be present in 54% of all subjects. Among subjects with anodal capture, bipolar LBBAP resulted in diminished improvement of LV d*P*/d*t*_max_ when compared with unipolar LBBAP, confirming published work by Ali et al.^44^ In subjects with anodal capture, bipolar LBBAP shortened RV activation time by 14 ms compared with unipolar LBBAP; however, bipolar and unipolar LBBAP produced similar reductions in QRS duration. This observation is relevant for clinical practice and procedural planning. The use of an LBBAP lead in a position that does not allow programming of a unipolar pacing configuration, such as the atrial port or RV port of the currently existing CRT-D device, may diminish the potential for hemodynamic improvement. In the future, device manufacturers may develop resynchronization devices that allow unipolar pacing configurations in all designated channels potentially designed to accommodate conduction system pacing leads. The extent to which anodal capture impacts the long-term outcome of LBBAP remains to be determined. Mechanistically, the impact of anodal capture in patients with a diseased left conduction system can be perceived as programmed DSP. In such cases, RV activation from the anode may dominate over the latent left bundle conduction activated from the cathode, and the septum will be nonphysiologically activated from right to left rather than left to right.

### Limitations

Our study population favored IVCD subjects and may, therefore, not be representative of the general CRT population. The relatively small study size compromised statistical power during subgroup analysis. The study was also limited to reporting only acute responses. A good predictive acute measure for CRT response remains to be established. Paced QRS-duration shortening is correlated with long-term mortality reduction.^45–47^ Acute invasive hemodynamic measurements (eg, LV d*P*/d*t*_max_) may be predictive of improved outcomes. In a multicenter prospective randomized controlled trial, LVcv lead placement guided by invasive LV d*P*/d*t*_max_ measurements demonstrated better rates of reverse remodeling, albeit data on mortality are lacking.^48^ Further, the present study excluded patients with complete atrioventricular conduction block, a population that may also benefit from LOT-CRT because atrioventricular conduction block precludes fused CRT delivered by specific algorithms. Another potential limitation of this study was the absence of diastolic function assessment. There is limited evidence that normalization of ventricular activation by His-Purkinje conduction system pacing should be associated with improved diastolic function.^29,49^ The long-term outcome of any resynchronization strategy will depend on its impact on contractile force and cardiac relaxation properties. Finally, classification of LBBB versus IVCD is somewhat subjective and should not be considered a dichotomy, because both conditions may coexist.

### Conclusions

This prospective, multicenter, acute hemodynamic study demonstrated that LOT-CRT decreased QRS duration more than LBBAP and BVP. LOT-CRT increased LV d*P*/d*t*_max_ similarly to BVP and more than LBBAP alone. Compared with patients with LBBB, those with IVCD experienced less QRS shortening during resynchronization pacing but similar improvements in LV d*P*/d*t*_max_. The incremental value of LOT-CRT over LBBAP on LV d*P*/d*t*_max_ was most pronounced in subjects with a baseline QRS ≥171 ms and in subjects with DSP only. Finally, in a CRT cohort with intact atrioventricular conduction, anodal capture during bipolar LBBAP resulted in a diminished LV contractile force and earlier RV activation but no change in QRS duration when compared with unipolar LBBAP.

## ARTICLE INFORMATION

### Acknowledgments

This study was sponsored by Medtronic plc. The authors acknowledge the trial and data management staff at Medtronic, especially Rachel Rose, Joy Aso, Fabio Pradella, and Jessica Mikacevich.

### Sources of Funding

This is a Medtronic-sponsored study.

### Disclosures

Dr Jastrzębski: speaker/consultant honoraria from Abbott, Biotronik, Boston Scientific, and Medtronic. Dr Foley: consultant to Medtronic, proctor for Medtronic. Dr Chandrasekaran: honoraria, consultancy fees and funding support from Medtronic, Biotronik, and Abbot. Dr Whinnett: speaker honoraria, consulting fees, and institutional fellowship/research support from Abbot, Boston Scientific, and Medtronic. Dr Vijayaraman: Medtronic: honoraria, consultant, research and fellowship support Abbott: honoraria, consultant Boston Scientific/Biotronik: honoraria; patent: HBP delivery tool. Dr Upadhyay: consulting and speaker honoraria from Abbott, Biotronik, Boston Scientific, GE Healthcare, Medtronic, Philips, Rhythm Science, and Zoll Medical. R.D. Schaller: speaking honoraria for Medtronic. Dr Richardson: consulting and speaking honoraria from Medtronic, Inc. Dr Herweg: fellowship support from Medtronic, speaker for Medtronic and Abbott.

Dr Stadler, D. Kudlik, R. Waxman, Dr Zimmerman, J. Burrell, and Dr Cornelussen are Medtronic employees. The other authors report no conflicts

### Supplemental Material

Figures S1–S3

## Supplementary Material


