# Supplementary material for: Multicenter Hemodynamic Assessment of the LOT-CRT Strategy: When Does Combining Left Bundle Branch Pacing and Coronary Venous Pacing Enhance Resynchronization?: Primary Results of the CSPOT Study
Source: Circ Arrhythm Electrophysiol. 2024 Oct 23;17(11):e013059. doi: 10.1161/CIRCEP.124.013059 (PMC11575906; doi:10.1161/CIRCEP.124.013059)
Supplement: Supplementary file 1 [file hae-17-e013059-s001.pdf]

SUPPLEMENTAL MATERIAL

Supplemental Figures:

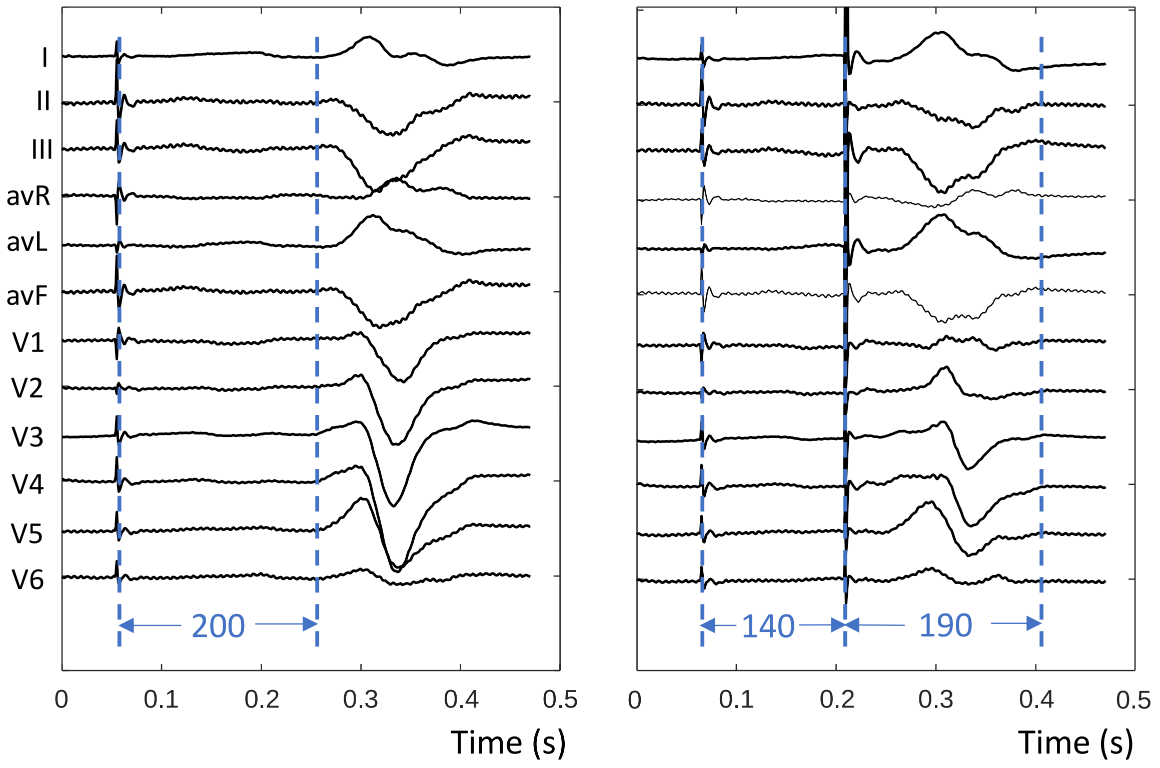

Supplemental Figure S1: Determination of default AV-delay and calculation of paced QRS-duration. Left: The interval between an atrial pace and the earliest QRS activation on the 12 lead ECG (200ms) was multiplied by 70% to determine a default AV delay (140ms). Right: Unipolar LBBAP at the default AV delay of 140ms. Each pacing intervention was tested at 5 AV delays (Default + 60, Default +30, Default, Default -30, Default – 60). QRS duration was measured from the pacing artifact to the latest termination of the QRS complex in all 12 leads (190ms in this case).

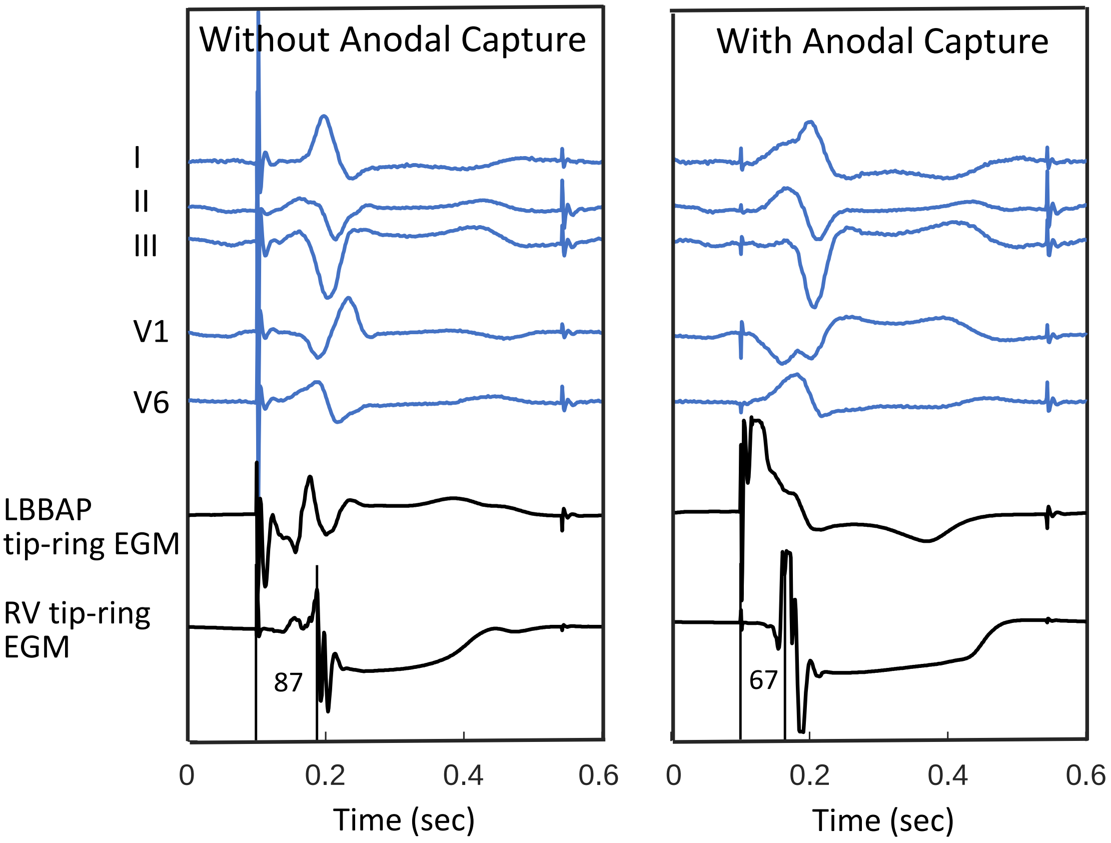

Supplemental Figure S2: Anodal capture determination. Example of absence (left) and presence (right) of anodal capture during LBBAP in the same patient. The RV tip-ring EGM shows earlier RV activation with anodal capture (numbers are expressed in ms). LBBAP = left bundle branch area pacing.

Panel A (top)
Panel B (bottom)

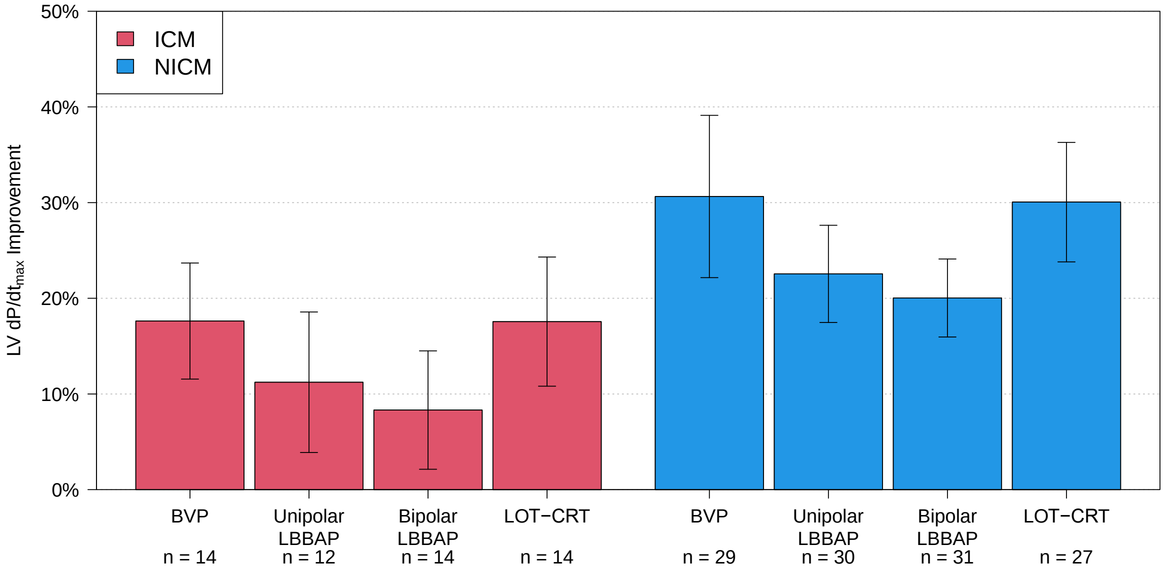

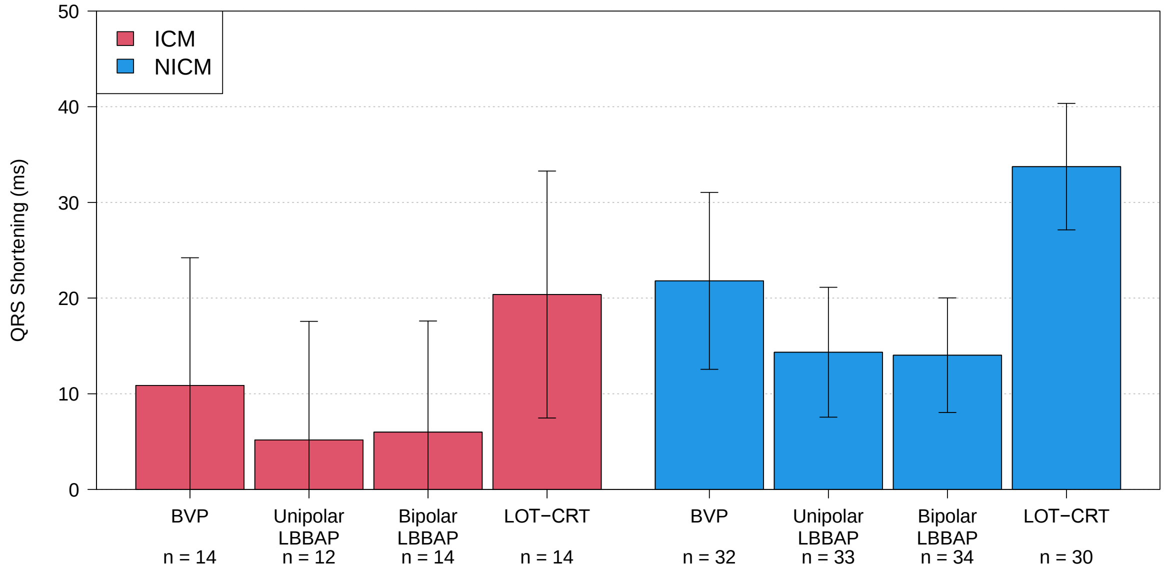

Supplemental Figure S3: Subclassification according to ischemic cardiomyopathy. PANEL A: LV dP/dtmax increase (% from baseline), comparison between groups: p = 0.004, comparison of relative effects of pacing configurations: p = 0.95 and PANEL B: QRS-duration shortening (absolute decrease from baseline) classified per Ischemic or non-ischemic cardiomyopathy, comparison between groups: p = 0.06, comparison of relative effects of pacing configurations: p = 0.81. LBBAP = left bundle branch

area pacing, including deep septal pacing. BVP = biventricular pacing. LOT-CRT = Left bundle optimized therapy.
